# Supplementary material for: COZOID: contact zone identifier for visual analysis of protein-protein interactions
Source: BMC Bioinformatics. 2018 Apr 6;19:125. doi: 10.1186/s12859-018-2113-6 (PMC5889581; doi:10.1186/s12859-018-2113-6)
Supplement: Supplementary file 2 — Software build. Executable binary file of the software tool. (ZIP 118,784 kb) [file 12859_2018_2113_MOESM2_ESM.zip › cozoid/java/docs/beansbinding-1.2.1-doc.zip!/org/jdesktop/beansbinding/BindingListener.html]

BindingListener (Beans Binding)


---


|  |  |  |
| --- | --- | --- |
| **Overview** \| **Package** \| **Class** \| **Use** \| **Tree** \| **Deprecated** \| **Index** \| **Help** | |  |
| **PREV CLASS**   **NEXT CLASS** | **FRAMES**    **NO FRAMES**     **All Classes** |  |
| SUMMARY: NESTED \| FIELD \| CONSTR \| METHOD | DETAIL: FIELD \| CONSTR \| METHOD |  |


---


## org.jdesktop.beansbinding Interface BindingListener

**All Superinterfaces:**: java.util.EventListener

**All Known Implementing Classes:**: AbstractBindingListener

---

``` public interface BindingListener extends java.util.EventListener ```

`BindingListeners` are registered on `Bindings` or `BindingGroups`
to listen for changes to the state of `Bindings`

**See Also:**: `Binding`, `BindingGroup`

---

| **Method Summary** | |
| --- | --- |
| `void` | `bindingBecameBound(Binding binding)`             Notification that a `Binding` has been bound. |
| `void` | `bindingBecameUnbound(Binding binding)`             Notification that a `Binding` has been unbound. |
| `void` | `sourceChanged(Binding binding, PropertyStateEvent event)`             Notification that the source property of a `Binding` has fired a `PropertyStateEvent` for the `Binding's` source object. |
| `void` | `synced(Binding binding)`             Notification that the source and target of a `Binding` have been made in sync. |
| `void` | `syncFailed(Binding binding, Binding.SyncFailure failure)`             Notification that the `Binding` attempted to sync the source and target, but the sync failed. |
| `void` | `targetChanged(Binding binding, PropertyStateEvent event)`             Notification that the target property of a `Binding` has fired a `PropertyStateEvent` for the `Binding's` target object. |

**Method Detail**

### bindingBecameBound

```
void bindingBecameBound(Binding binding)
```

:   Notification that a `Binding` has been bound.

    :   **Parameters:**: `binding` - the `Binding`

---


### bindingBecameUnbound

```
void bindingBecameUnbound(Binding binding)
```

:   Notification that a `Binding` has been unbound.

    :   **Parameters:**: `binding` - the `Binding`

---


### syncFailed

```
void syncFailed(Binding binding,
                Binding.SyncFailure failure)
```

:   Notification that the `Binding` attempted to sync the source and
    target, but the sync failed.

    :   **Parameters:**: `binding` - the `Binding`: `failure` - the reason the sync failed

---


### synced

```
void synced(Binding binding)
```

:   Notification that the source and target of a `Binding` have
    been made in sync.

    :   **Parameters:**: `binding` - the `Binding`

---


### sourceChanged

```
void sourceChanged(Binding binding,
                   PropertyStateEvent event)
```

:   Notification that the source property of a `Binding` has fired
    a `PropertyStateEvent` for the `Binding's` source object.

    :   **Parameters:**: `binding` - the `Binding`

---


### targetChanged

```
void targetChanged(Binding binding,
                   PropertyStateEvent event)
```

:   Notification that the target property of a `Binding` has fired
    a `PropertyStateEvent` for the `Binding's` target object.

    :   **Parameters:**: `binding` - the `Binding`


---


|  |  |  |
| --- | --- | --- |
| **Overview** \| **Package** \| **Class** \| **Use** \| **Tree** \| **Deprecated** \| **Index** \| **Help** | |  |
| **PREV CLASS**   **NEXT CLASS** | **FRAMES**    **NO FRAMES**     **All Classes** |  |
| SUMMARY: NESTED \| FIELD \| CONSTR \| METHOD | DETAIL: FIELD \| CONSTR \| METHOD |  |


---
